# Identification of an Unclassified Paramyxovirus in *Coleura afra*: A Potential Case of Host Specificity

**DOI:** 10.1371/journal.pone.0115588

**Published:** 2014-12-31

**Authors:** Gael D. Maganga, Mathieu Bourgarel, Judicael Obame Nkoghe, Nadine N'Dilimabaka, Christian Drosten, Christophe Paupy, Serge Morand, Jan Felix Drexler, Eric M. Leroy

**Affiliations:** 1 Centre International de Recherches Médicales de Franceville (CIRMF), Franceville, Gabon; 2 Institut National Supérieur d'Agronomie et de Biotechnologies (INSAB), Franceville, Gabon; 3 Centre de Coopération Internationale en Recherche Agronomique pour le Développement (CIRAD), UPR AGIRs, Montpellier, France; 4 Centre de Coopération Internationale en Recherche Agronomique pour le Développement (CIRAD), UPR AGIRs, Harare, Zimbabwe; 5 Institut de Recherche pour le Développement (IRD), UMR 224 (MIVEGEC), IRD/CNRS/UM1/UM2, Montpellier, France; 6 Institute of Virology, University of Bonn Medical Centre, Bonn, Germany; 7 des Sciences de l'Evolution, CNRS-UM2, CC065, Université de Montpellier 2, Montpellier, France; University of Pretoria, South Africa

## Abstract

Bats are known to harbor multiple paramyxoviruses. Despite the creation of two new genera, *Aquaparamyxovirus* and *Ferlavirus*, to accommodate this increasing diversity, several recently isolated or characterized viruses remain unclassified beyond the subfamily level. In the present study, among 985 bats belonging to 6 species sampled in the Belinga caves of Gabon, RNA of an unclassified paramyxovirus (Belinga bat virus, BelPV) was discovered in 14 African sheath-tailed bats (*Coleura afra*), one of which exhibited several hemorrhagic lesions at necropsy, and viral sequence was obtained in two animals. Phylogenetically, BelPV is related to *J virus* and *Beilong virus* (BeiPV), two other unclassified paramyxoviruses isolated from rodents. In the diseased BelPV-infected *C. afra* individual, high viral load was detected in the heart, and the lesions were consistent with those reported in wild rodents and mice experimentally infected by *J virus*. BelPV was not detected in other tested bat species sharing the same roosting sites and living in very close proximity with *C. afra* in the two caves sampled, suggesting that this virus may be host-specific for *C. afra*. The mode of transmission of this paramyxovirus in bat populations remains to be discovered.

## Introduction

Members of the Paramyxoviridae family are pleomorphic enveloped viruses [1] divided into two subfamilies, *Paramyxovirinae* and *Pneumovirinae*. *Paramyxovirinae* has recently been subdivided into seven genera: *Aquaparamyxovirus*, *Avulavirus*, *Ferlavirus, Henipavirus*, *Morbillivirus*, *Respirovirus*, and *Rubulavirus* (http://ictvonline.org/virusTaxonomy.asp?version=2012). Viruses of this family affect a wide range of animals, including primates, birds, carnivores, ungulates, snakes, cetaceans and humans, and cause a wide variety of infections, such as measles, mumps, pneumonia and encephalitis in humans, and distemper, peste des petits ruminants, Newcastle disease and respiratory tract infections in animals. However, several paramyxoviruses (PVs) have not been classified into any of these seven genera, including *Nariva virus* (NarPV) [2], *Mossman virus* (MosPV) [3], *Beilong virus* (BeiPV) [4], *J virus* (JPV) [5], [6], *Tupaia paramyxovirus* (TupPV) [7] and *Tailam virus* [8], all of which belong to a group of novel paramyxoviruses isolated from wild animals, as well as *Salem virus* isolated from horses [9]. Among them, only JPV has been shown to be pathogenic, causing extensive haemorrhagic lesions in rodents [6]. Horizontal transmission is the principal mode of intraspecies PV infection, suggesting that contaminated faeces, urine or saliva may be responsible for spillover to other species [10].

Bats have a close evolutionary relationship with several genera of mammalian paramyxoviruses [11]. Otherwise, bat-borne paramyxoviruses are in close relationship to known paramyxoviruses of mammalian. These small mammals are known to harbour a broad diversity of PVs, including emergent henipaviruses (Nipah virus and Hendra virus) and rubulaviruses [Menangle virus, Tioman virus, Mapuera virus, and Tuhoko virus 1, 2 and 3 (ThkPV-1, ThkPV-2 and ThkPV-3)]. A very broad diversity of paramyxoviruses, including Henipa-, Rubula-, Pneumo- and Morbilli-related viruses, have been detected in six of ten tested bat families [11]. Whereas most of the viruses identified in bats do not seem to cause clinical disease in these animals, there have been reports of rabid bats [12], [13] and of unusually large numbers of animals succumbing to infection by rabies virus [14].

As part of a large-scale investigation of viral diversity in bats and of associated zoonotic risks, we have previously detected a bat paramyxovirus in one insectivorous African sheath-tailed bat (*Coleura afra*) [11], exhibiting several hemorrhagic lesions at necropsy. We therefore examined occurrence of this bat paraymxovirus in other bats.

## Materials and Methods

### Ethics statements

The study was conducted in the Belinga mountains (northeast Gabon), where Ebola outbreaks occurred in 1994–1996 and 2001–2002. All the capture events, animal handling, euthanasia and transfer of samples across country borders were performed in accordance with the guidelines of the American Society of Mammalogists (http://www.mammalsociety.org/committees/animal-care-and-use) [15]: bats were captured following recommendations by Kunz and Parsons [16] and identified by trained field biologist. Captured bats were removed carefully from nets as soon as possible to minimize injury, drowning, strangulation, or stress. Safe and humane euthanasia was achieved through the use of inhalant anaesthetic (halothane) prior to autopsy.

All work (capture, euthanasia and autopsy) was carried out with authorization from the Gabonese Ministry of Water and Forestry (Département de la Faune et de la Chasse – Authorization N°003/MEFEPA/SG/DGEF/DFC and N°0021/MEFEPA/SG/DGEF/DFC) and the Gabonese Ministry of Higher Education, Scientific Research and Innovation (Centre National de la Recherche Scientifique et Technique – Authorization N° AR0027/10/MENESRI/CENAREST/CG/CST/CSAR).

### Bat sampling

Samples of liver, spleen, kidney, lung, heart, gut, brain and salivary glands were collected, stored in liquid nitrogen and transferred to the CIRMF laboratory (Centre International de Recherches Médicales de Franceville, Gabon), where they were stored at −80°C until analysis. Blood samples were also collected, except from the smallest individuals (body mass <12 g). A total of 985 bats (Table 1) were sampled from the caves of Faucon (1°07′N, 13°20′E), Zadié (0°98′N, 13°19′E) and Batouala (0°82′N, 13°45’E) in July 2009, December 2009 and June 2010.

**Table 1:** Overview of specimens collected in Belinga caves and tested by specific BelPV qPCR assay.

Sampling sites	Bats species	Total	Positive RT-PCR
Batouala cave	*Coleura afra*	26	6
	*Hipposideros* cf. ruber	117	0
	*Hipposideros gigas*	59	0
	*Miniopterus inflatus*	161	0
	*Rousettus aegyptiacus*	18	0
			
Faucon cave	*Coleura afra*	68	8
	*Hipposideros* cf. ruber	281	0
	*Hipposideros gigas*	121	0
	*Miniopterus inflatus*	10	0
	*Rhinolophus alcyone*	14	0
			
Zadié cave	*Rousettus aegyptiacus*	110	0
			
**Total**		**985**	**14**
			
Belinga caves	***Diptera: Nycteribiidae***		
	*Eucampsipoda africana*	51	0
	*Nycteribia schmidlii scotti*	26	0
	*Penicillidia fulvida*	21	0
			
	***Diptera: Streblidae***		
	*Brachytarsina allaudi*	3	0
	*Raymondia huberi huberi*	1	0
			
	***Diptera: Culicidae***		
	*Uranotaenia nigromaculata*	10	0
	*Culex wigglesworthi*	320	0
			
Total		**432**	**0**

Interestingly, at autopsy, only one *C. afra* out of the 26 individuals, from Batouala cave, exhibited diarrhea and severe hemorrhagic lesions in both thoracic and abdominal organs, along with lung congestion and pleurisy.

### Viral PCR screening

Virological screening of the diseased bat was performed to identify the cause of these lesions. The screening process included the whole Paramyxoviridae family and also filoviruses [Marburg virus (MARV) and Zaire Ebola virus (EBOV)], given the nature of the lesions and the known filovirus tropism for bats. Briefly, approximately 100 mg each of this animal's liver and spleen were pooled and crushed in 600 µl of cold PBS in a ball-mill tissue grinder (Genogrinder 2000, Spex Centripep). Total RNA was extracted using a Biorobot EZ1 and the EZ1 RNA tissue mini kit (Qiagen) according to the manufacturer's guidelines. The RNA was then tested for paramyxoviruses, using three heminested reverse transcription-PCR (hnRT-PCR) assays targeting the polymerase gene [17], and also for Marburg virus [18] and Zaire Ebola virus [19]. However, the screening was extended to Arenaviruses, Flaviviruses, Alphaviruses, polyomaviruses, Orthopoxviruses, Parapoxviruses, Influenza viruses, Lyssaviruses and Rhabdoviridae family.

### Paramyxovirus detection in bat populations

To further investigate the presence of the virus in bat populations, a strain-specific real-time RT-PCR assay (primers: GB09-478-F, 5′-GGCGGCTCTTAAAAGTGAATG-3′; GB09-478-R, 5′-GCGGGGTCAAATTGGTCAT-3′; probe: GB09-478-P, 5′-TCCAGCACAAACATATCCGAGAAGGCTAG-3′) was designed within the initial PCR fragment and was used to test total RNA extracted from mixed liver and spleen samples from each of all the other bat species. The amplification was performed in a final volume of 25 µl, containing, 12.5 µl TaqMan^R^ 2X PCR Master Mix (Applied Biosystems), 0.5 µl each primer and probe (10 µM), 1 µl bovine serum albumin (1 µg/µl) (Invitrogen), 5 µl cDNA and RNAse-free water (Invitrogen). Amplification generally involved 2 min at 55°C, 10 min at 95°C followed by 45 cycles of 15s at 95°C and 1 min at 58°C.

### Virus distribution in organs of infected bats

In order to determine the organ distribution of this virus in infected bats, total RNA was extracted from heart, liver, spleen, kidney, lung, intestine and brain samples from all 14 real-time RT-PCR-positive bats, as described previously, and screened, using the same strain-specific real-time RT-PCR assay shown above.

### Entomological study

Transmission by hematophagous arthropods was studied. Hematophagous arthropods were collected inside Faucon and Zadié caves. Mosquitoes were sampled using CDC light traps whereas bat-flies were manually collected on bats (*Coleura afra*, *Miniopterus inflatus*, *Hipposideros* cf. *ruber* and *Rousettus aegyptiacus*) trapped in the Faucon and Zadié caves between November and December 2009, June 2010, and January, March and April 2011. After the morphologic species determination, insects were crushed by monospecific pools (up to 10 specimens for mosquitoes, and between 1 and 5 for bat-flies) in 300 µl PBS. Total RNA was extracted using 100 µl of the supernatant from each pool by using the RNeasy Mini Kit (Qiagen) and then was tested with the specific real-time RT-PCR assay described above.

### Blast and phylogenetic analysis

The viral sequences obtained were first compared to those available in the public database using the algorithm "Blastn" from BLAST program [20] and then aligned with homologous sequences of *Paramyxoviridae* reference strains from GenBank, using the MEGA program version 5 [21]. Bayesian inference of phylogeny was done using MrBayes V.3.2 software and the GTR+G+I nucleotide substitution model [22] for two million generations with a burn-in of 25%.

## Results

### Identification and molecular characterization

One of three hnRT-PCR, Respiro-, Morbilli- and Henipaviruses PCR (RMH-PCR) assays yielded an amplicon of 559 base pairs (bp) from diseased bat, in collaborative work with the Bonn Institute of Virology Bonn (Germany). The PCR product was sequenced with dye terminator chemistry (Applied Biosystems). No other virus was detected in the diseased bat.

**Figure 1:**
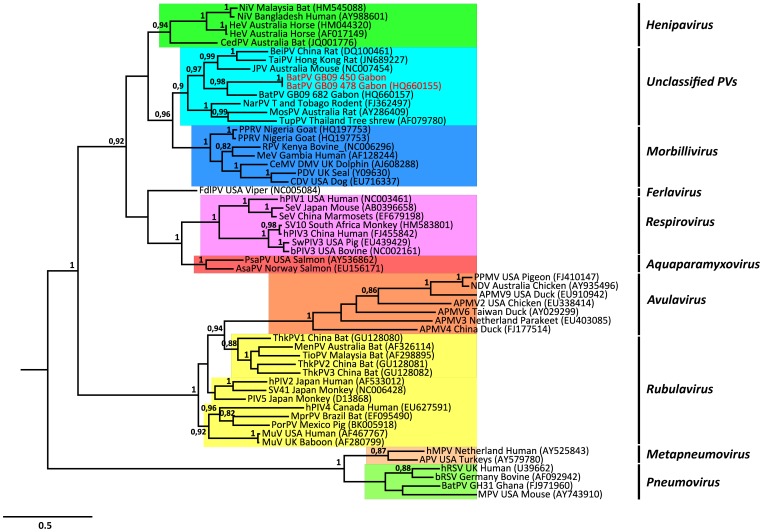
Phylogenetic tree based on a 439-basepair fragment of the polymerase gene (L) of members of the *Paramyxoviridae* family. Sequences generated in this study are highlighted in red. Bayesian posterior probabilities are shown; values <0.80 were removed for clarity. The viruses are designated as follows (virus abbreviation/typical host/accession numbers of reference sequences in brackets): HeV  =  Hendra virus, NiV  =  Nipah virus, BatPV  =  Bat paramyxovirus, BeiPV  =  Beilong virus, JPV  = J virus, MosPV  =  Mossman virus, TupPV  =  Tupaia paramyxovirus, NarPV  =  Nariva virus, PDV  =  Phocine distemper virus, CDV  =  Canine distemper virus, CeMV DMV  =  Cetacean morbillivirus strain dolphin morbillivirus, MeV  =  Measles virus, PPRV  =  Peste-des-petits ruminants virus, RPV  =  Rinderpest virus, FdlPV  =  Fer-de-lance virus, PSPV  =  Pacific salmon paramyxovirus, ASPV  =  Atlantic salmon paramyxovirus, SeV  =  Sendai virus, bPIV3  =  Bovine parainfluenza virus 3, hPIV1  =  Human parainfluenza virus 1, hPIV3  =  Human parainfluenza virus 3, SwPIV3  =  Swine parainfluenza virus 3, NDV  =  Newcastle disease virus, PigeonPMV  =  Pigeon paramyxovirus, AMPV9  =  Avian paramyxovirus type 9, AMPV6  =  Avian paramyxovirus type 6, AMPV2  =  Avian paramyxovirus type 2, AMPV3  =  Avian paramyxovirus type 3, AMPV4  =  Avian paramyxovirus type 4, PIV5  =  parainfluenza virus 5, SV41  =  Simian virus 41, MenPV  =  Menangle paramyxovirus, MprPV  =  Mapuera virus, MuV  =  Mumpsvirus, PorPV  =  Porcine rubulavirus, TioPV  =  Tioman paramyxovirus, hPIV2  =  Human parainfluenza virus 2, hMPV  =  Human metapneumovirus, MPV  =  Murine pneumonia virus, bRSV  =  Bovine respiratory syncytial virus, hRSV  =  Human respiratory syncytial virus, APV  =  Avian Pneumovirus, ThkPV-1  =  Tuhoko virus 1, ThkPV-2  =  Tuhoko virus 2, ThkPV-3  =  Tuhoko virus 3.

Blast and phylogenetic analysis confirmed that the sequence from diseased bat, designated BatPV GB09 478 (Genbank accession number HQ660155), belonged to the Paramyxoviridae family. It showed 65% and 66% nucleotide identity, respectively, with JPV and BeiPV, and had pairwise nucleotide identities of 36–42% with the Henipavirus group and 37–40% with the Morbillivirus group. Phylogenetic analysis indicated that this bat PV, that we named Belinga bat virus (BelPV), clustered with unclassified paramyxoviruses and was located between the genera *Morbillivirus* and *Henipavirus* (Fig. 1).

In this study, among all animals tested by the strain-specific real-time RT-PCR assay only 14 *C. afra* bats, including the diseased animal, were positive. Bat samples positive by specific real-time RT-PCR were then tested with RMH-hemi nested-PCR for molecular characterization. Another 439-bp PCR product was amplified from one liver-spleen pool from a second *C. afra* specimen, and was sequenced. These two phylogenetically related sequences (Fig. 1), designated BatPV GB09 478 and BatPV GB09 450, displayed 100% of nucleotide identity.

### Prevalence and organ distribution of BelPV in *Coleura afra*

BelPV-specific RNA was detected only in *C. afra*, with a prevalence of 14.9% (14/94 of the *C. afra* individuals sampled from the Faucon and Batouala caves) (Table 1). Interestingly, no bats belonging to the other five species tested positive.

**Figure 2:**
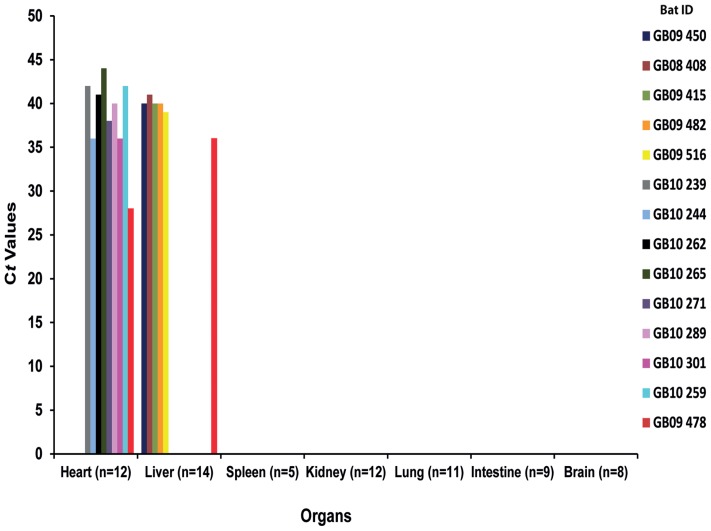
Virus distribution in organs from *Coleura afra* individuals. Virus distribution is shown in terms of C*t* values on the y-axis for each bat organ tested (x-axis). *n* represents the number of organs available for the study.

BelPV RNA was detected in the heart (8/12) and liver (5/14), at low and variable loads (Table 2 and Fig. 2). No significant difference in the BelPV RNA detection rate was found between the two organs (χ^2^ = 2.476, df = 1, p>0.20). In the bat exhibiting severe hemorrhagic lesions (GB09 478), both the liver and heart were BelPV-positive, with a higher viral load in the heart (C*t* value  = 28).

**Table 2:** Results of real-time PCR on organs from *Coleura afra* individuals.

Samples ID	Heart	Liver	Spleen	Kidney	Lung	Intestine	Brain
GB09 408	ND	41	ND	ND	ND	ND	Undet.
GB09 415	Undet.	40	ND	Undet.	Undet.	ND	ND
GB09 450	Undet.	40	ND	Undet.	ND	ND	ND
GB09 478	28	36	ND	Undet.	Undet.	Undet.	ND
GB09 482	ND	40	ND	Undet.	Undet.	Undet.	ND
GB09 516	Undet.	39	ND	Undet.	Undet.	ND	ND
GB10 239	42	Undet.	Undet.	Undet.	Undet.	Undet.	Undet.
GB10 244	36	Undet.	Undet.	Undet.	Undet.	Undet.	Undet.
GB10 262	41	Undet.	ND	Undet.	Undet.	Undet.	Undet.
GB10 265	44	Undet.	ND	ND	ND	ND	ND
GB10 271	38	Undet.	Undet.	Undet.	Undet.	Undet.	Undet.
GB10 289	40	Undet.	Undet.	Undet.	Undet.	Undet.	Undet.
GB10 301	36	Undet.	ND	Undet.	Undet.	Undet.	Undet.
GB10 259	42	Undet.	Undet.	Undet.	Undet.	Undet.	Undet.

Numbers indicate the cycle threshold (C*t*). ND, not done because of missing samples.

Undet, C*t* undetermined.

### Entomological study

In total, 432 arthropods were collected inside Faucon and Zadié caves, including Culicidae (10 Uranotaenia nigromaculata and 320 Culex wigglesworthi), Nycteribiidae (51 Eucampsipoda africana, 26 Nycteribia schmidlii scotti and 21 Penicillidia fulvida) and Streblidea (Brachytarsina allaudi and 1 Raymondia huberi huberi) (Table 1). No arthropods tested were positive for Belinga Bat Virus (Table 1).

## Discussion

The BelPV nucleotide sequence obtained showed similarity with the JPV and BeiPV sequences. BelPV has previously been reported to hold a phylogenetic position between the genera *Henipavirus* and *Morbillivirus*. The same phylogenetic position had been observed with MosPV and J-V [4].

In this study, organs with high BelPV concentrations are different from those found with high paramyxovirus concentrations in pteropodids and microchiroptera bats. In microchiroptera bats from Brazil, spleen has been found more positive than the others organs with highest viral load, as in *Eidolon helvum* (megachiroptera bat) in Africa [11]. However, in our study majority of spleen were not available.

The within-host BelPV distribution tended to be organ-specific. BelPV seemed to be restricted to the heart and liver. In contrast, JPV has been isolated from blood, lung, liver, kidney and spleen of experimentally infected laboratory mice [6] but not in heart. The BelPV distribution for the heart and liver, together with the high viral load in heart tissue, could suggest that this virus is likely to be present in the bloodstream and might thus be transmitted during aggressive contacts between bats, or by blood-sucking vectors. Nethertheless, viremia was not proven. BelPV RNA was not searched from blood because in these small species of bats blood was difficult to collect in the field.

We detected BelPV only in *Coleura afra* and not in other bat species sharing the same roosting sites and living in very close proximity in the two caves sampled. However, it has been shown that bats of different species occupying the same roosting sites can share the same viruses. Marburg virus had been detected in *Rousettus aegyptiacus* and *Hipposideros* sp. bats living in Kitaka cave in Uganda [23] and *Miniopterus inflatus* and *Rousettus aegyptiacus* bats caught in Goroumbwa mine in the Democratic Republic of the Congo [24]. These bat species are known to live in close proximity. Thus, virus transmission between different bat species is possible [25]. Thus, we can speculate that the failure to detect BelPV in other bat species sharing the same caves would suggest that this virus has strong host specificity for *C. afra*, as well as restricted intraspecies transmission. Henipaviruses occur naturally in fruit bats belonging to the genus *Pteropus* [26], and this also appears to be true of severe acute respiratory syndrome-like coronaviruses in *Rhinolophus* bats [27], [11].

In view of our data we can assume that BelPV might have pathogenic potential for its host *C. afra*. Indeed, high viral load was detected in the heart of the diseased bat, and the lesions were consistent with those reported in wild rodents and mice experimentally infected by JPV [6].

Although BelPV RNA was also detected in asymptomatic bats, pathogenicity may appear in long term under some immunological and/or ecological conditions. Indeed, virus must not induce pathology to persist or adapt within its reservoir host. Many authors suggested that persistence in the absence of pathology or disease appears to be a common characteristic of bat viruses in their natural host population [28], [29]. However, a severe immunodepression for instance, may increase the risk of infection with opportunistic pathogens. Under some environmental conditions (cool environments for example), some avirulent pathogens, such as *Geomyces destructans*, causative agent of white-nose syndrome, may become pathogenic in hibernating bats in North America [30], [31]. Nevertheless, infection by BelPV may be mild for bats and thus the pathology observed not directly related. Otherwise, it may also be that this animal had an underlying disease or infection with a different pathogen. Even in this case, we might not draw any conclusions neither establish a link with lesions seen. Therefore, the pathogenicity of the BelPV should be demonstrated by experimental animal infection. Otherwise, viral antigens or RNAs should be detected histologically in the lesions of naturally-infected bats. However, the unavailability of biological tissues from the diseased bat failed to perform these analyzes. Consequently, other captures of *Coleura afra* species are considered in order to find BelPV again for further studies (pathogenicity to its host, isolation and complete genome characterization). However, *Coleura afra* is a migratory species living in colonies of several hundred individuals. In Gabon, this species, which has been recently described, is not present all year round in the caves of the north-east of the country, making the studies on this species difficult and thereof partly explaining the lack of virological studies.

Some viruses appear to cause clinical disease in wild-living bats; these include lyssaviruses and an ebola-like filovirus named Lloviu virus [32], [33], [34].

Bats are the natural reservoirs for many viruses, including emerging zoonotic viruses such as SARS-CoV [27], Hendra and Nipah viruses [26], [35], Ebola virus [36], Marburg virus [23], [37], rabies virus and other Lyssaviruses [11]. In general, humans are infected through an intermediate amplifying host such as palm civets for SARS-CoV, horses for Hendra virus and pigs for Nipah virus [38]. However, in humans Nipah virus outbreaks linked to bats exposure have been reported [39]. It remains to be shown whether the BelPV reported here presents a zoonotic risk. Nonetheless, like most RNA viruses, for example coronaviruses, characterized by high mutation and/or recombination rates [40], PVs may adapt to novel hosts, including humans. A serological test capable of detecting antibodies to this virus in human populations living in the vicinity of these animals is needed to assess zoonotic potential.

All the blood-sucking arthropods collected from bats, as well as mosquitoes collected in the caves where bat sampling took place, were negative for BelPV, in keeping with the lack of known PV vectors [41]. However, BelPV transmission by blood-sucking vectors within the Gabonese population of *C. afra* cannot be ruled out. Indeed, a haemosporidian parasite (*Polychromophilus*) was found in a blood parasite vector (*Penicillidia fulvida*) in Faucon cave in Gabon in 1977 [42] and also in its host *M. inflatus* (greater long-fingered bat) from the same cave in 2010 and 2011 [43]. In addition, the methodology used to collect flying hematophagous insects (based on light traps) possibly introduced a bias by selecting only those attracted by light. Therefore, we can not exclude that additional sampling techniques could increase the number of mosquitoes species or groups known to colonize caves such as sandflies or biting midges. Hence, the natural mode of transmission of this unclassified paramyxovirus in bat populations, through bat-bat aggression for example, remains to be determined.

This association between *C. afra* and BelPV could serve as an interesting model, (i) to evaluate modes of transmission within host populations, (ii) to study host-virus interactions (pathogenesis and host specificity), and (iii) to evaluate the zoonotic risk of a newly identified virus.

Further studies of *C. afra* populations and a broader diversity of arthropod vectors, spanning larger areas and time scales, are needed to confirm this apparent host-virus specificity, and to determine the modes of BelPV transmission. Further studies are needed to characterize complete BelPV genome and demonstrate the pathogenicity of this virus for its host *Coleura afra*.

## References

[1] Lamb RA, Kolakofsky D (2001) *Paramyxoviridae*: the viruses and their replication. In: Knipe DM, Howley PM, Griffin DE, Lamb RA, Martin MA, Roizman B, Straus SE (Eds.), Fields Virology, 4^th^ ed. Lippincott Williams and Wilkins, Philadelphia; pp. 1305–1340.

[2] Tikasingh ES, Jonkers AH, Spence L, Aitken TH (1966) Nariva virus, a hitherto undescribed agent isolated from the Trinidadian rat, *Zygodontomys b. brevicauda* (J.A. Allen and Chapman). *Am J Trop Med Hyg* 15:235–238. 4956232 10.4269/ajtmh.1966.15.235

[3] Campbell RW, Carley JG, Doherty R, Domrow R, Filippich C, et al (1977) Mossman virus, a paramyxovirus of rodents isolated in Queensland. *Search* 8:435–436.

[4] Li Z, Yu M, Zhang H, Magoffin DE, Jack PJM, et al (2006) Beilong virus, a novel paramyxovirus with the largest genome of non-segmented negative-stranded RNA viruses. *Virol* 346 219–228. 10.1016/j.virol.2005.10.039 16325221

[5] Jun MH (1976) Studies on a virus isolated from wild mice (*Mus musculus*). M. Sc. Thesis. James Cook University, Townsville, Queensland.

[6] Jun MH, Karabatsos N, Johnson RH (1977) A new mouse paramyxovirus (*J virus*). *Aust J Exp Biol Med Sci* 55:645–647. 614833 10.1038/icb.1977.63

[7] Tidona CA, Kurz HW, Gelderblom HR, Darai G (1999) Isolation and molecular characterization of a novel cytopathogenic paramyxovirus from tree shrews. *Virol* 258:425–434. 10.1006/viro.1999.9693 10366580

[8] Woo PC, Lau SK, Wong BH, Wong AY, Poon RW, et al (2011) Complete genome sequence of a novel paramyxovirus, *Tailam virus*, discovered in Sikkim rats. *J virol* 85(24):13473–4 doi:10.1128/JVI.06356-11. 22106385 PMC3233173

[9] Renshaw RW, Glaser AL, Vancampen H, Weiland F, Dubovi EJ (2000) Identification and phylogenetic comparison of *Salem virus*, a novel paramyxovirus of horses. *Virol* 270:417–429. 10.1006/viro.2000.0305 10793001

[10] Plowright RK, Field HE, Smith C, Divljan A, Palmer C, et al (2008) Reproduction and nutritional stress are risk factors for Hendra virus infection in little red flying foxes (*Pteropus scapulatus*). *Proc R Soc B* 275:861–869 10.1098/rspb.2007.1260) PMC2596896 18198149

[11] Drexler JF, Corman VM, Müller MA, Maganga GD, Vallo P, et al (2012) Bats host major mammalian paramyxoviruses. *Nat Commun* 3:796. 22531181 10.1038/ncomms1796 PMC3343228

[12] Fraser GC, Hooper PT, Lunt RA, Gould AR, Gleeson LJ, et al (1996) Encephalitis caused by a lyssavirus in fruit bats in Australia. *Emerg Infect Dis* 2:327–331. 8969249 10.3201/eid0204.960408 PMC2639915

[13] Johnson N, Phillpotts R, Fooks AR (2006) Airborne transmission of lyssaviruses. *J Med Microbiol* 55:785–79. 16687600 10.1099/jmm.0.46370-0

[14] Baer GM, Smith JS (1991) Rabies in nonhematophagous bats. *In* ‘‘The Natural History of Rabies’’, (G. M Baer, ed.), CRC Press, Boca Raton, FL, USA, pp.341–366.

[15] Gannon W, Sikes R (2007) Guidelines of the American Society of Mammalogists for the use of wild mammals in research. *Journal of Mammalogy* 88:809–823. 10.1093/jmammal/gyw078 PMC5909806 29692469

[16] Kunz TH, Parsons S (2009) Ecological and behavioral methods for the study of bats. 2nd ed. Kunz TH, Parsons S, editors Baltimore, Maryland: Johns Hopkins University Press.

[17] Tong S, Chern SW, Li Y, Pallansch MA, Anderson LJ (2008) Sensitive and broadly reactive reverse transcription-PCR assays to detect novel paramyxoviruses. *J Clin Microbiol* 46:2652–2658. 18579717 10.1128/JCM.00192-08 PMC2519498

[18] Towner JS, Pourrut X, Albarino CG, Nkogue CN, Bird BH, et al (2007) Marburg virus infection detected in a common African bat. *PLoS ONE* 2:e764. 17712412 10.1371/journal.pone.0000764 PMC1942080

[19] Towner JS, Sealy TK, Khristova ML, Albarino CG, Conlan S, et al (2008) Newly discovered ebola virus associated with hemorrhagic Fever outbreak in Uganda. *PLoS Pathog* 4:e1000212. 19023410 10.1371/journal.ppat.1000212 PMC2581435

[20] Altschul S, Gish W, Miller W, Myers E, Lipman D (1990) "Basic local alignment search tool". *Journal of Molecular Biology* 215 (3):403–410 doi:10.1016/S0022-2836(05)80360-2. 2231712

[21] Tamura K, Peterson D, Peterson N, Stecher G, Nei M, et al (2011) MEGA5: Molecular Evolutionary Genetics Analysis using Maximum Likelihood, Evolutionary Distance and Maximum Parsimony Methods. Molecular Biology and Evolution 28:2731–2739. 21546353 10.1093/molbev/msr121 PMC3203626

[22] Huelsenbeck JP, Ronquist F (2001) MRBAYES: Bayesian inference of phylogenetic trees. *Bioinformatics* 17:754–5. 11524383 10.1093/bioinformatics/17.8.754

[23] Towner JS, Amman BR, Sealy TK, Carroll SAR, Comer JA, et al (2009) Isolation of Genetically Diverse Marburg Viruses from Egyptian Fruit Bats. *PLoS Pathog* 5:e1000536 doi:10.1371/journal.ppat.1000536. 19649327 PMC2713404

[24] Swanepoel R, Smit SB, Rollin PE, Formenty P, Leman PA, et al (2007) Studies of Reservoir Hosts for Marburg Virus. *Emerg Infect Dis* 13:1847–51 10.3201/eid1312.071115). 18258034 PMC2876776

[25] Chu DK, Peiris JS, Chen H, Guan Y, Poon LL (2008) Genomic characterizations of bat coronaviruses (1A, 1B and HKU8) and evidence for co-infections in *Miniopterus* bats. *J Gen Virol* 89(5):1282–1287. 18420807 10.1099/vir.0.83605-0

[26] Halpin K, Young PL, Field HE, Mackenzie JS (2000) Isolation of Hendra virus from pteropid bats: a natural reservoir of Hendra virus. *J Gen Virol* 81:1927–1932. 10900029 10.1099/0022-1317-81-8-1927

[27] Li W, Shi Z, Yu M, Ren W, Smith C, et al (2005) Bats are natural reservoirs of SARS-like coronaviruses. *Science* 310:676–9. 16195424 10.1126/science.1118391

[28] Calisher CH, Childs JE, Field HE, Holmes KV, Schountz T (2006) Bats: important reservoir hosts of emerging viruses. *Clin Microbiol Rev* 19:531–45. 16847084 10.1128/CMR.00017-06 PMC1539106

[29] Chu DK, Poon LL, Guan Y, Peiris JS (2008) Novel astroviruses in insectivorous bats. *J Virol* 82:9107–9114. 18550669 10.1128/JVI.00857-08 PMC2546893

[30] Blehert DS, Hicks AC, Behr MJ, Meteyer CU, Berlowski-Zier BM, et al (2009) Bat white-nose syndrome: an emerging fungal pathogen? *Science* 323:227 doi:10.1126/science.1163874. 18974316

[31] Warnecke L, Turner JM, Bollinger TK, Lorch JM, Misra V, et al (2012) Inoculation of bats with European *Geomyces destructans* supports the novel pathogen hypothesis for the origin of white-nose syndrome. *Proc Natl Acad Sci USA* 109:6999–7003 doi:10.1073/pnas.1200374109. 22493237 PMC3344949

[32] McColl KA, Chamberlain T, Lunt RA, Newberry KM, Middleton D, et al (2002) Pathogenesis studies with Australian bat lyssavirus in grey- headed flying foxes (Pteropus poliocephalus). *Aust Vet J* 80:636–641. 12465817 10.1111/j.1751-0813.2002.tb10973.x

[33] Banyard AC, Hayman D, Johnson N, McElhinney L, Fooks AR (2011) Bats and Lyssaviruses. *Advances in Virus Research*, 79. doi: doi:10.1016/B978-0-12-387040-7.00012-3. 21601050

[34] Negredo A, Palacios G, Vázquez-Morón S, González F, Dopazo H, et al (2011) Discovery of an Ebolavirus-Like Filovirus in Europe. *PLoS Pathog* 7(10):e1002304 doi:10.1371/journal.ppat.1002304. 22039362 PMC3197594

[35] Johara MY, Field H, Rashdi AM, Morrissy C, Heide Bvd, et al (2001) Nipah virus infection in bats (Order Chiroptera) in Peninsular Malaysia. *Emerging Infectious Diseases* 7:439–441. 11384522 10.3201/eid0703.010312 PMC2631791

[36] Leroy EM, Kumulungui B, Pourrut X, Rouquet P, Hassanin A, et al (2005) Fruit bats as reservoirs of Ebola virus. *Nature* 438:575–576. 16319873 10.1038/438575a

[37] Maganga GD, Bourgarel M, Ebang Ella G, Drexler JF, Gonzalez JP, et al (2011) Is Marburg Virus Enzootic in Gabon? *J Infect Dis* 204 (Suppl 3)S800–3. 21987754 10.1093/infdis/jir358

[38] Wong S, Lau S, Woo P, Yuen KY (2007) Bats as a continuing source of emerging infections in humans. *Rev Med Virol* 17:67–91. 17042030 10.1002/rmv.520 PMC7169091

[39] Montgomery JM, Hossain MJ, Gurley E, Carroll DS, Croisier A, et al (2008) Risk factor for Nipah virus encephalitis in Bangladesh. *Emerg Infect Dis* 14:1526–32. 18826814 10.3201/eid1410.060507 PMC2609878

[40] Lau SK, Woo PC, Li KS, Huang Y, Tsoi HW, et al (2005) Severe acute respiratory syndrome coronavirus-like virus in Chinese horseshoe bats. *Proc Natl Acad Sci USA* 102:14040–14045. 16169905 10.1073/pnas.0506735102 PMC1236580

[41] Frank GH (1981) *Paramyxovirus* and *Pneumovirus* diseases of animals and birds: comparatives aspects and diagnosis. *In* Kurstack Eeditors: *Comparative diagnosis of viral diseases*, New York, Academic Press, pp. 187–233.

[42] Landau I, Chabaud AG, Miltgen F, Baccam D (1980) *Dionisia bunoi* n. g. n. sp., Haemoproteidae parasite du microchiroptère *Hipposideros cyclops* au Gabon. *Ann Parasitol Hum Comp* 55:271–280. 6773461 10.1051/parasite/1980553271

[43] Duval L, Mejean C, Maganga GD, Makanga BK, Mangama Koumba LB, et al (2012) The chiropteran haemosporidian *Polychromophilus melanipherus*: A worldwide species complex restricted to the family *Miniopteridae* . *Infections Genetics and Evolution* 12:1558–1566. 10.1016/j.meegid.2012.06.006 22721902

